# Methylprednisolone intravenous pulse therapy for pediatric patients with post-infectious bronchiolitis obliterans: an update

**DOI:** 10.36416/1806-3756/e20230373

**Published:** 2024-04-08

**Authors:** Silvia Onoda Tomikawa, Joaquim Carlos Rodrigues, Cleyde Miryam Aversa Nakaie, Luiz Vicente Ribeiro Ferreira da Silva

**Affiliations:** 1. Divisão de Pneumologia Pediátrica, Instituto da Criança, Hospital das Clínicas, Faculdade de Medicina, Universidade de São Paulo (SP) Brasil.

## TO THE EDITOR:

Post-infectious bronchiolitis obliterans (PIBO) is a rare form of chronic obstructive lung disease that follows a lower respiratory tract infection and results in partial or complete obliteration of the small airways.^1^ ^,^ ^2^

The use of corticosteroids is the most common anti-inflammatory therapy for PIBO; nonetheless, there has been no controlled trials to prove its efficacy and safety.^1^ ^,^ ^2^ High-dose methylprednisolone intravenous pulse therapy has been proposed to enhance the therapeutic effect and reduce side effects of prolonged corticosteroid therapy.^1^ ^,^ ^3^

We reported our first experience in children with PIBO treated with corticosteroid pulse therapy between 1996 and 2007.^4^ The objective of the current study is to describe our experience with this approach in the subsequent period (2007-2019).

We included PIBO patients attending our outpatient clinic (≤ 18 years of age) who were treated with monthly intravenous high-dose methylprednisolone pulse (MP) therapy cycles (30 mg/kg body weight/day for three consecutive days) between 2007 and 2019. The diagnosis of PIBO was given upon a combination of clinical and radiological findings; none of the patients was submitted to lung biopsy. Clinical criteria included signs of persistent and severe airway obstruction that persisted for over six weeks following a severe acute respiratory infection in a previously healthy child, excluding other chronic obstructive lung diseases. Radiological criteria were based on HRCT findings, including significant air trapping, mosaic pattern, and/or bronchiectasis.

Data were obtained from the medical records and included the number of acute wheezing attacks and hospitalizations within the six-month period before treatment, and at 6, 12, 18, and 24 months thereafter. Description of growth, the need of supplemental oxygen, and chronic oral corticosteroid usage, as well as lung function results and MP therapy-related adverse effects were also registered when available. The nonparametric ANOVA (Friedman test) with post hoc Dunnett’s test was performed to compare wheezing attacks and hospitalization frequencies between periods; anthropometric comparisons used t-test for paired samples. Statistical analysis was performed using the IBM SPSS Statistics software package, version 23.0 (IBM Corporation, Armonk, NY, USA). Statistical significance defined as α = 5%. This study was approved by the research ethics committee of the institution (CAPPesq HCFMUSP, protocol no. 0695/07).

A total of 17 patients (9 boys = 53%) diagnosed between 10-59 months of age (median = 22 months) were included in the study. The interval between the onset of the disease and diagnosis was 1-28 months (median = 10 months).

While the presumed etiology was post-infectious, most of the referring hospitals did not perform studies of viral etiology and therefore only two patients had a previous result of an adenovirus infection.

The most common radiological finding was mosaic perfusion pattern (in 88%), followed by atelectasis (in 59%), bronchiectasis (in 35%), bronchial wall thickening (in 41%), alveolar filling (in 17%), air trapping (in 17%) and Swyer-James-MacLeod syndrome (in 12%).

The median number of MP cycles was 9 (range, 5-17). The median age at MP start was 23 months (range, 10-60 months), and the median interval between the onset of disease and MP therapy was 11 months (range, 2-28 months). In addition, all 17 patients were given inhaled corticosteroids with long-acting β_2_-agonist bronchodilators during the entire follow-up period. Other treatments included therapy for gastroesophageal reflux (n = 8; 47%), azithromycin (n = 9; 52%) and anti-hypertensive drugs (n =3; 17%). 

The median numbers of acute wheezing attacks and hospitalizations were significantly reduced after starting MP therapy when compared with the previous period (p = 0.001 and p < 0.001, respectively). Half of the patients were able to discontinue supplemental oxygen use, and most of those who remained dependent were able to step down the flow or duration needed. We also observed improvement of growth in the first year after MP therapy started (Table 1).

**Table 1:** Clinical and laboratory evaluation before and after methylprednisolone pulse therapy (n = 17)

Variable	Before	After	Time needed to discontinue
Prolonged oral corticosteroid therapy	8 patients (47%)	0 patient	4.5 months (range, 2-13 months)
Domiciliary oxygen therapy			
Total	13 patients (76%)	7 patients (41%)	11 months (range, 8-13 months)
Full-time	10 patients	1 patient	
Overnight	1 patient	6 patients	
Anthropometric measurements	Before	After one year	p
Length for age (mean Z score)	−1.37	−0.84	0.016
Weight for age (mean Z score)	−0.70	−0.19	0.20
BMI (mean Z score)	0.09	0.49	0.25

Spirometry results were available during follow-up in 9 patients (median age = 6.0 years; range, 5.5-8.1 years), and depicted moderate-to-severe obstructive disease with reduced vital capacity-median [IQR] in % of predicted values: FEV_1_% = 41 [40-42]; FEV_1_/FVC% = 76 [67.8-80.0]; and FEF_25-75%_ = 22 [19-26]-and no significant response to bronchodilators.^5^

Safety of MP therapy was overall very good, and adverse effects were transient and of minor concern. No patient was diagnosed with arterial hypertension after starting MP therapy, and no one was diagnosed with diabetes mellitus in the follow-up. Few patients were actively screened for long-term effects of steroid therapy such as fundoscopy, urinary tract lithiasis, or bone density, but results were normal in those investigated.

We started MP therapy with corticosteroids for PIBO cases some years ago, aiming to maximize therapeutic effect and reduce side effects of prolonged systemic corticosteroid use. However, we had no reference to judge how long the treatment should be kept for, and which patients would benefit the most. We now believe that treatment for some patients was longer than necessary. After learning from the results of our initial study,^4^ we established that if the patient shows no improvement after 6 cycles of MP therapy, it should be discontinued, and we also agreed to reduce the total number of cycles, preferably up to 12 months of treatment.

In this current study, 17 children with PIBO treated with MP therapy presented with significant clinical improvement: fewer wheezing exacerbations and hospitalizations, and improved oxygen saturation. This accumulated experience allowed us to establish that the most important factors to indicate MP therapy for BO patients should be dependence of chronic systemic corticosteroid therapy or the need for home oxygen therapy, as well as prolonged or frequent hospitalizations due to respiratory impairment.

An additional aspect that needs to be considered is the lag before starting the treatment. In our previous study,^4^ the median interval between the onset of the disease and the start of MP therapy was 18.5 months. In the current study, this interval was 11 months. Presumably, the effectiveness of anti-inflammatory treatment would be reduced if the initial event has taken place too long before its start, due to airway fibrosis. A retrospective and controlled study^6^ evaluated the responsiveness of 17 patients with PIBO who were treated with MP therapy and the responder group had a significantly shorter median interval between the initial episode (4 months) and the start of MP therapy than did the non-responder group (50 months), indicating that MP therapy works better when indicated earlier.

Regarding the side effects, all acute effects observed during the methylprednisolone intravenous infusion were transient and not considered serious in our series. Even linear growth of the patients showed to improve, possibly due to clinical stabilization and discontinuation of chronic oral use of corticosteroids.

We conclude that MP therapy may be an effective and safe strategy to improve the clinical course of PIBO but to be used for shorter periods of time than we previously recommended.
